# Effect of Xanthii Fructus alcohol extract on proliferation and apoptosis of HFLS-RA and its mechanism

**DOI:** 10.1097/MD.0000000000032541

**Published:** 2022-12-30

**Authors:** Hai Jiang, Huan Yu, Senwang Zheng, Xuejiao Wang, Ajiao Hou, Haixue Kuang, Liu Yang

**Affiliations:** a Key Laboratory of Chinese Materia Medica, Heilongjiang University of Chinese Medicine, Ministry of Education, Harbin, China.

**Keywords:** apoptosis, HFLS-RA, NF-κB signaling pathway, proliferation, Wnt/β-catenin signaling pathway, Xanthii fructus

## Abstract

Xanthii fructus (XF) is the dried and mature fruit of Xanthium sibiricum Patr. It has the effects of anti-inflammatory, antioxidant and anti-arthritic. Rheumatoid arthritis (RA) is the most common inflammatory disorder and often leads to disability. However, there are few studies on the treatment of RA by XF and the specific mechanism of treatment has not been clarified. This study was designed to explore the effects of proliferation and apoptosis by XF on human fibroblast-like synovial-RA (HFLS-RA) cells and investigate its mechanism. The cell proliferation ability was detected by MTS assay. Hoechst 33,342 staining was used to detect apoptosis, and the apoptosis rate was detected by flow cytometry. The expression levels of NF-κB p65 and β-catenin were detected by Western Blotting. MTS, Hoechst 33,342, flow cytometry analysis showed that the alcohol extract of XF inhibited human fibroblast-like synovial-RA cells proliferation and promoted apoptosis in a dose-dependent manner. Western Blotting experiment showed that the extract of XF could reduce the expression levels of NF-κB p65 and β-catenin. The extract of XF has a significant therapeutic effect on RA in vitro by regulating NF-κB signaling pathway and Wnt/β-catenin signaling pathway. Our research will help to clarify the potential pharmacological mechanism of XF on RA and provide experimental basis for the application of XF in clinical treatment.

## 1. Introduction

rheumatoid arthritis (RA) is a chronic inflammatory disease characterized by multi-joint, symmetrical, and aggressive joint inflammation of the small joints of the hands and feet.^[1,2]^ It is mainly caused by excessive synovial tissue hyperplasia, pannus formation, and erosion of cartilage. The typical pathological features include joint swelling, pain, morning stiffness, and deformity, often accompanied by joint organ involvement.^[3,4]^ These diseases have seriously affected the daily lives of patients and caused trauma to their physical and mental health. Therefore, the treatment of RA can relieve patients’ pain and improve their quality of life.

During the investigation of RA, it was found that FLS cells were 1 of the main cell types in the synovial tissues of patients. The human fibroblast-like synovial-RA (HFLS-RA) cells have become the most popular cell line for most researchers to study RA.^[5]^ Up to now, the comprehensive pathogenesis of RA is still unclear. A large number of studies have shown that NF-κB proteins and β-catenin play an important role in the inflammatory response, cell proliferation, and apoptosis of RA.^[6–8]^ The NF-κB proteins, which belong to multifunctional nuclear transcription factors, are abundant in FLS-RA cells.^[9]^ When NF-κB is stimulated and activated, its inhibitory protein IκB is dissociated and detached, allowing NF-κB to enter the nucleus to regulate inflammatory factors such as TNF-α, IL-1β, COX2, and chemokines.^[10]^ β-catenin is the key downstream protein of the Wnt classical pathway.^[11]^ After its stable accumulation, β-catenin can enter the nucleus and initiate the transcription of downstream genes to promote cell adhesion and activation.^[12]^ Studies have shown that the Wnt/β-catenin signaling pathway is reflected in regulating excessive proliferation and inflammation of FLS cells in RA.^[13]^ Hence, NF-κB protein and β-catenin play a key role in RA, and through research, it has been found that the inhibition of these 2 proteins will contribute to the treatment of RA, and can be used as a key point in the study of RA.

The treatment of RA is aimed at reducing the inflammatory reaction of joints, inhibiting the development of lesions and irreversible bone destruction. Nowadays, the main drugs used in clinics include non-steroidal anti-inflammatory drugs, slow-acting antirheumatic drugs, immunosuppressants, and immune and biological agents, among others. However, these drugs are costly and mainly have severe adverse reactions, such as cardiovascular and gastrointestinal bleeding, liver and kidney toxicity, growth inhibition, infection, and tumor risk.^[14]^ To solve these problems, the development of new natural products is critical for RA therapy. Traditional Chinese medicine has the synergistic effect of multi-component, multi-target, and multi-pathway, and has less toxic and side effects. It is reported that its main pharmacological effects are related to pain relief, improvement of inflammation, regulation of immune function, protection of cartilage, reduction of pannus formation, and inhibition of synovial hyperplasia, among others.^[15,16]^

**Figure 1.**
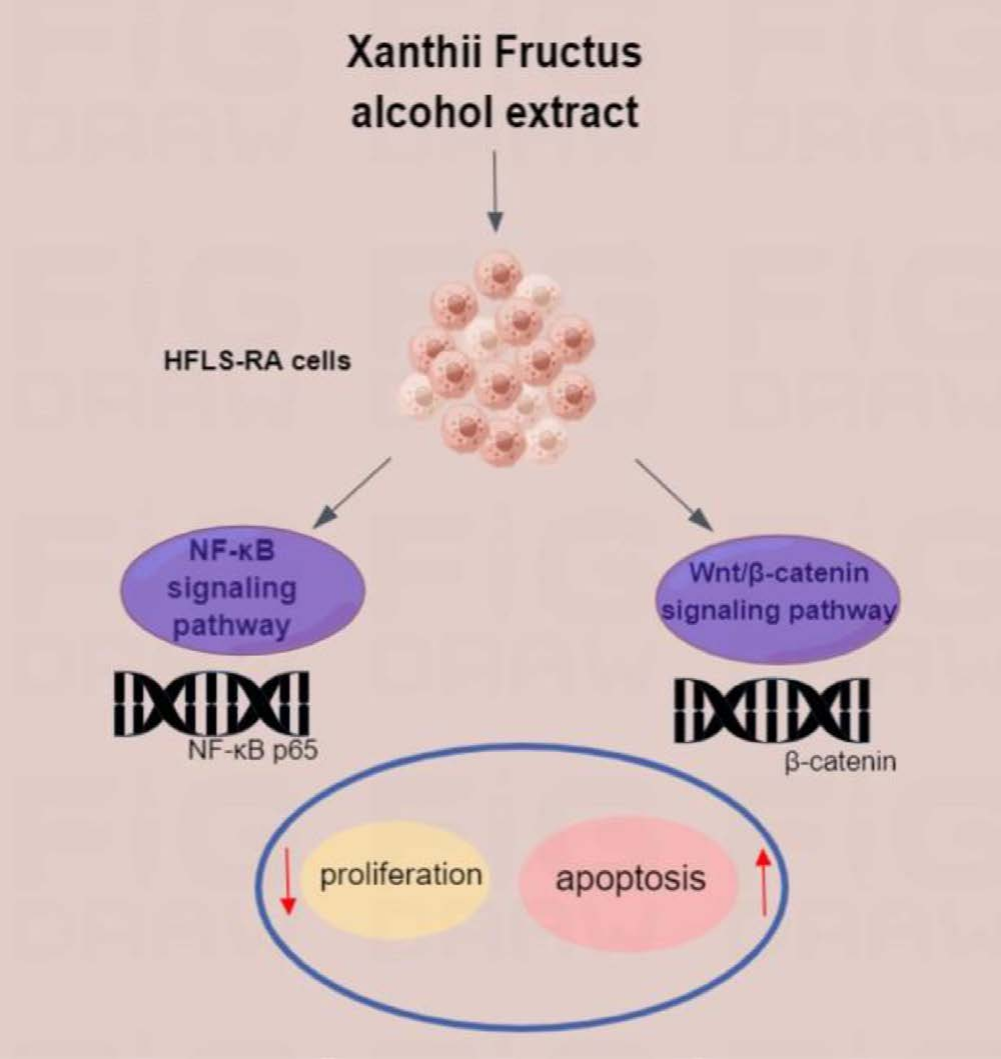
Graphic abstract diagram.

XF, also called “cangerzi” in China, has effects of antibacterial, anti-inflammatory, analgesic, antiallergic, antioxidant, antitumor and immunomodulatory.^[17]^ It is mainly used for treating cold, sinusitis, rheumatic arthralgia, rubella pruritus and other diseases. Researchers discovered early that XF can be used for external treatment of RA.^[18]^ After that, it was proved by animal experiments that XF has a therapeutic effect on RA, and achieved good curative effect.^[19,20]^ However, the mechanism of its therapeutic effect needs further study. We researched the curative effect of XF on RA by HFLS-RA cells and investigated relevant mechanisms. In order to provide an experimental basis for the rational application of XF in RA clinical treatment and further excavate its pharmacological mechanism. The graphic abstract of the article is shown in Figure 1.

## 2. Materials and methods

### 2.1. Main reagents and instruments

Dulbecco’s modified eagle medium (DMEM; Gibco, 8121267); (fetal bovine serum; ExCell Bio, FSP500); Penicillin-Streptomycin Solution (Cytiva, SV30010); 96-well plates and 6-well plates (Nest Biotechnology, 701001, 703001); CellTiter 96 AQueous 1 Solution Cell Proliferation Assay (MTS; G3580, Promega); Hoechst 33,342 (Solarbio, C0031); PBS (Solarbio, P1020); Annexin V-fluorescein isothiocyanate (FITC)/propidium iodide (PI) (Annexin V-FITC/PI) apoptosis detection kit (Vazyme, A211-01); BeyoGel™ Plus Precast PAGE Gel for Tris-Gly System (P0468S, Beyotime Biotechnology); TBS with Tween-20 (TBST; ST673, Beyotime Biotechnology); RIPA Lysis Buffer (P0013B, Beyotime Biotechnology); SDS-PAGE Sample Loading Buffer (P0015L, Beyotime Biotechnology); nitrocellulose (NC) membrane (FFN08, Beyotime Biotechnology) (phenylmethanesulfonyl fluoride; ST506, yotime Biotechnology); BCA Protein Assay Kit (CWBIO, CW0014S); SDS-PAGE Electrophoresis Buffer with Tris-Gly (Tris-Glycine SDS Buffer; P0014D, Beyotime Biotechnology); Tris-Glycine Transfer Buffer (P0572, Beyotime Biotechnology); NF-κB p65/RelA Mouse mAb (ABclonal,A10609); β-catenin Rabbit pAb (ABclonal,A11512); GAPDH Rabbit pAb (ABclonal, AC001); IRDye® 800CW Goat anti-Rabbit IgG (H + L)(Bioss, bs-40295G-IRDye8); IRDye® 800CW Goat anti-Mouse IgG (H + L)(Bioss, bs-40296G-IRDye8).

Microplate reader (BioTek Epoch); Fluorescence microscope (OLYMPUS); Flow cytometry (BD Biosciences); Odyssey CLx Imager Dual-color infrared imaging system (LI-COR).

### 2.2. Ethanol extracts of plant material

Dried and ripped XF were bought from Bozhou’s herbal market (Anhui Province, China) and were stockpiled at the Heilongjiang University of Chinese Medicine. A representative specimen (XF20181010BZ) was deposited in the laboratory and plant materials were authenticated by Prof Su Lianjie from the Heilongjiang University of Chinese Medicine, Harbin, China. The XF sample and 50% ethanol were heated and refluxed twice with a solid-liquid ratio of 1:10, each time for 2 hours, and filtered each time. The filtrates were combined and concentrated under reduced pressure to remove the ethanol solvent. Then, the solution was freeze-dried under vacuum. The dried powder was obtained, redissolved, and dispersed in DMEM. The concentration of the high dose was set at 5mg/mL, the middle dose at 4mg/mL, and the low dose at 3 mg/mL.

### 2.3. Culture for HFLS-RA cells

HFLS-RA cells were purchased from Otwo Biotech (ShenZhen) Inc. (HTX1835). They were cultured in DMEM supplemented with 10% fetal bovine serum and 1% penicillin-streptomycin solution (complete medium) at 37ºC and 5% CO2 air.

### 2.4. MTS assay

HFLS-RA cells were cultured overnight in a 96-well plate with complete medium. Then, the cells were incubated with XF (3, 4, 5 mg/mL) for 24 hours. Finally, 20 μL MTS (3-[4,5-dimethylthiazol-2-yl] -5-[3-carboxymethoxyphenyl]-2-[4-sulfophenyl]-2H-tetrazolium) was added to the 96-well plate. The absorbance at 490 nm was measured by a microplate reader (BioTek Epoch). The cell proliferation rate is expressed as the ratio (%) compared to the control group.

### 2.5. Hoechst 33,342 to measure apoptosis

HFLS-RA cells were cultured overnight in a 6-well plate with complete medium. Then, the cells were incubated with XF (3, 4, 5 mg/mL) for 24 hours. After the treatment, the Hoechst 33,342 (Solarbio, C0031) was used in accordance with the manufacturer’s instructions to determine apoptosis status. After washing with PBS for 2 to 3 times, the cells were detected and photographed by fluorescence microscope (Olympus, Tokyo, Japan).

### 2.6. Flow cytometry to measure apoptosis

HFLS-RA cells were incubated for 24 hours with XF at concentrations of 3, 4, and 5 mg/mL. As recommended by the manufacturer of the Annexin V-Fluorescein Isothiocyanate/Propidium Iodide (V-FITC/PI) Apoptosis Detection Kit (Vazyme, A211-01), cells were resuspended with binding buffer and mixed with 5 *µ*L of Annexin V-FITC and PI. The percentages of apoptosis were determined by flow cytometry (BD Biosciences).

### 2.7. Western blot analysis

HFLS-RA cells were cultured overnight in a 6-well plate with complete medium. Then, the cells were incubated with XF (3, 4, 5 mg/mL) for 24 hours. Finally, RIPA buffer containing protease inhibitor phenylmethanesulfonyl fluoride (Beyotime, Shanghai, China) was used to lyse the cells, and centrifuged and the supernatant was collected. The protein concentration of the supernatant was detected by a BCA Protein Assay Kit (Beyotime, Shanghai, China). The appropriate protein concentration was adjusted with the lysate, and mixed with the loading buffer, equal concentrations of proteins were separated by SDS-PAGE and electro-transferred to a nitrocellulose membrane (NC, Beyotime, Shanghai, China, FFN08). The latter NC membranes were blocked with 5% nonfat milk for 1 hour with shaking. Then, the membranes were incubated with primary antibodies including NF-κB p65 (ABclonal, Wuhan, China, A10609), β-catenin (ABclonal, Wuhan, China, A11512) and GAPDH (ABclonal, Wuhan, China, AC001) overnight at 4 °C. The NC membranes were rinsed with TBST thrice and incubated with secondary antibodies for 1h at room temperature. The expression of GAPDH was used for normalizing the proteins expressions of different groups. Bands were detected using a 2-color infrared laser imaging system (Odyssey Clx Image Studio 3.1). The experiments were repeated 3 times.

### 2.8. Statistical analyses

Statistical software GraphPad Prism, version 9.0 (GraphPad Software, La Jolla, California) was used for plotting and statistical analysis. Data were the mean ± standard error of the mean. The significance of differences between groups was evaluated using Student’s *t* test; *P* < .05 was considered significant.

## 3. Results

### 3.1. XF inhibited the proliferation of HFLS-RA cells

**Figure 2.**
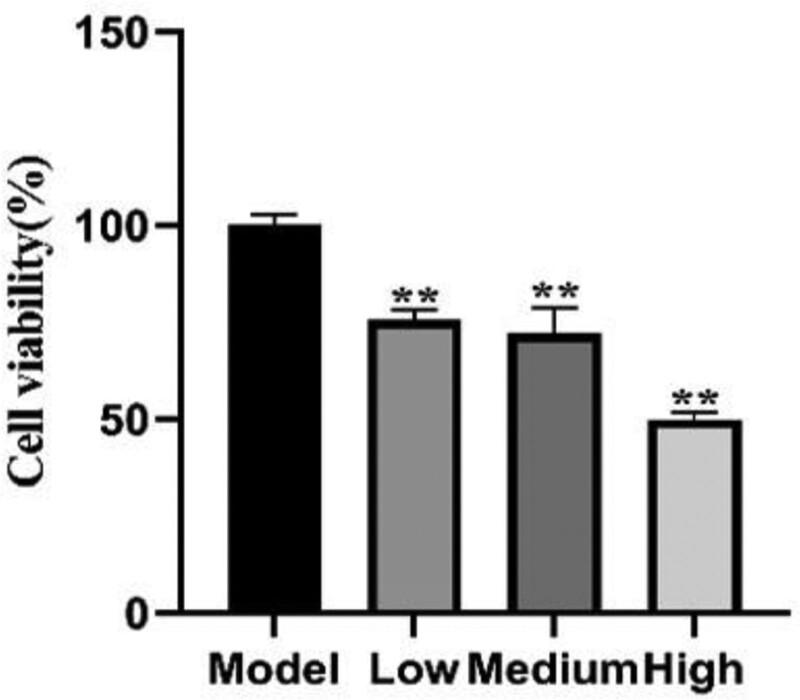
HFLS-RA cells were incubated with XF (3,4,5 mg/mL) for 24 hours and anti-proliferative effects of XF against HFLS-RA cells exhibited a concentration-dependent relationship. HFLS = human fibroblast-like synovial, RA = rheumatoid arthritis.

The effect of XF on the proliferation of HFLS-RA cells was considered with the MTS assay. XF (3, 4, 5 mg/mL) could significantly reduce the cell viability of HFLS-RA cells (*P* < .01, *P* < .01, *P* < .01), which was significantly lower than that of the untreated control group in a dose-dependent manner (Table 1). The results indicated that XF has a strong antiproliferative effect on HFLS-RA cells. The results are shown in Figure 2.

**Table 1:** OD value and survival rate of HFLS-RA cells of incubated with XF for 24 hours.

Group	OD value (24 h)	Cell survival rate (%)
Blank	0.1749 ± 0.01568	
Model	1.7705 ± 0.04882	100.00
Low	1.3393 ± 0.04548	75.65
Medium	1.2783 ± 0.11508	72.20
High	0.8812 ± 0.03376	49.77

HFLS = human fibroblast-like synovial, RA = rheumatoid arthritis, XF = Xanthii fructus.

### 3.2. XF induced the apoptosis of HFLS-RA cells

**Figure 3.**
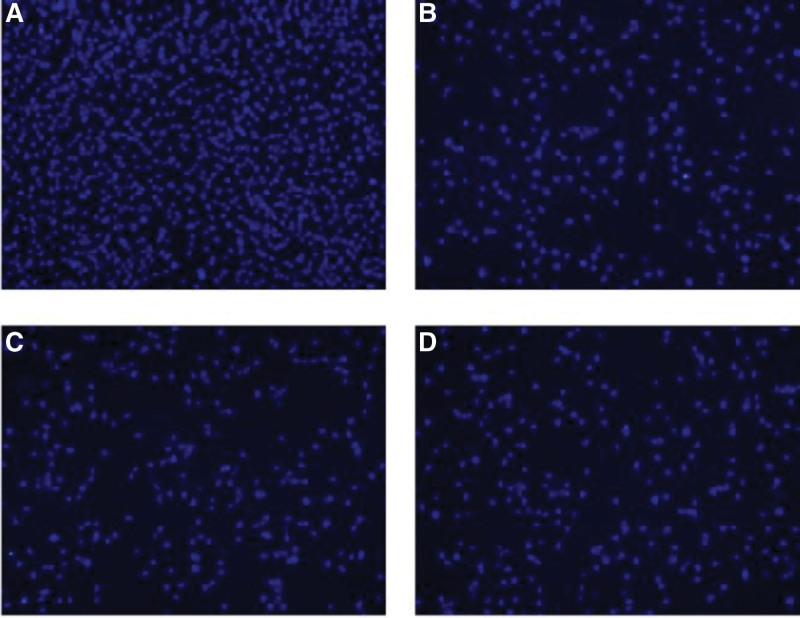
Hoechst 33,342 assay was used to investigate the effect of XF on the proliferation of HFLS-RA cells.

**Figure 4.**
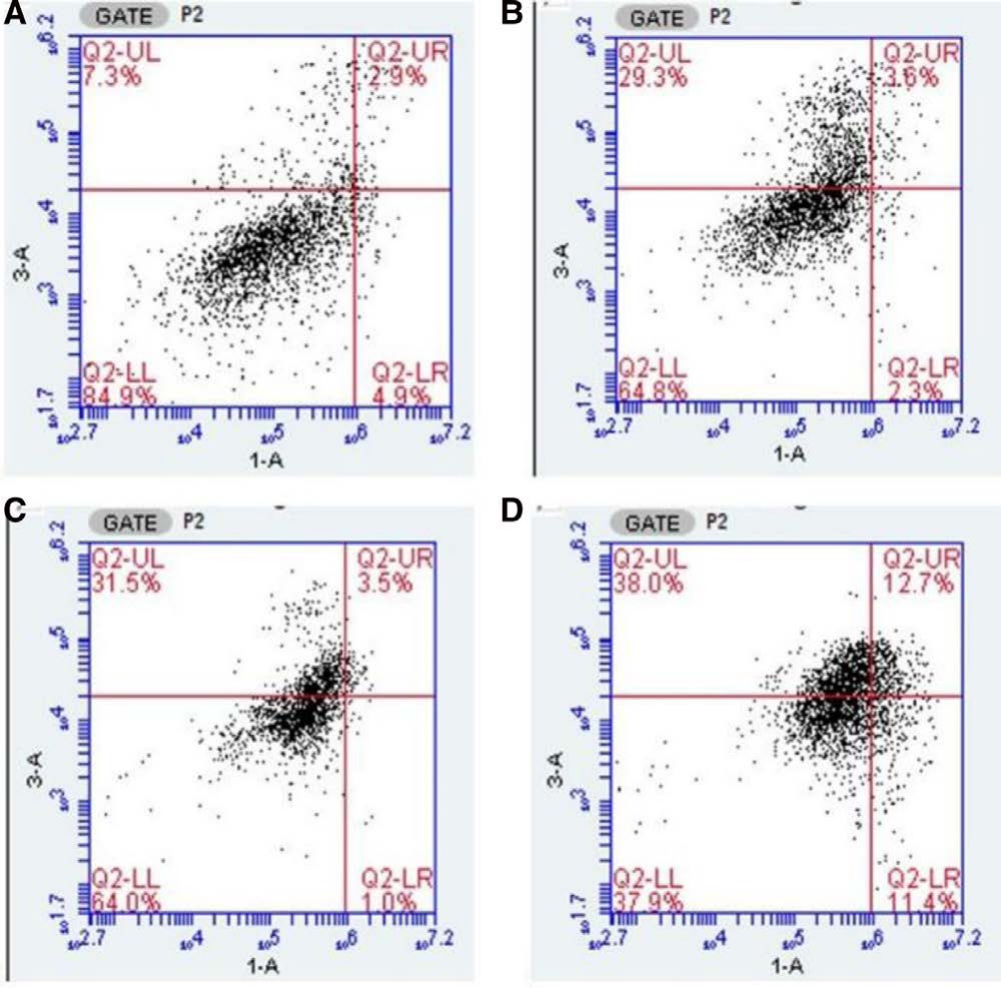
Percent apoptosis was determined by flow cytometry. Apoptotic cells were detected by staining with Annexin V-FITC/PI followed by flow cytometry. FITC = fluorescein isothiocyanate, PI = propidium iodide.

Hoechst 33,342 is a blue fluorescent dye that can penetrate the cell membrane and is often used for DNA staining. Compared to the control group, XF groups showed lower chromatin that was stained by Hoechst 33,342 in a dose-dependent manner. The blue staining in the nucleus of apoptotic cells can be observed by fluorescence microscopy (Fig. 3). To quantify the apoptotic rate, we next investigated the capacity of XF to increase the apoptosis of HFLS-RA cells by flow cytometry analysis. After detecting the apoptotic rate following treatment with XF, the results demonstrated that with an increase in concentration, the percentage of apoptosis also increased (Fig. 4). These 2 results both pointed to the fact that XF could induce the apoptosis of HFLS-RA cells.

### 3.3. Effect of XF on related proteins

**Figure 5.**
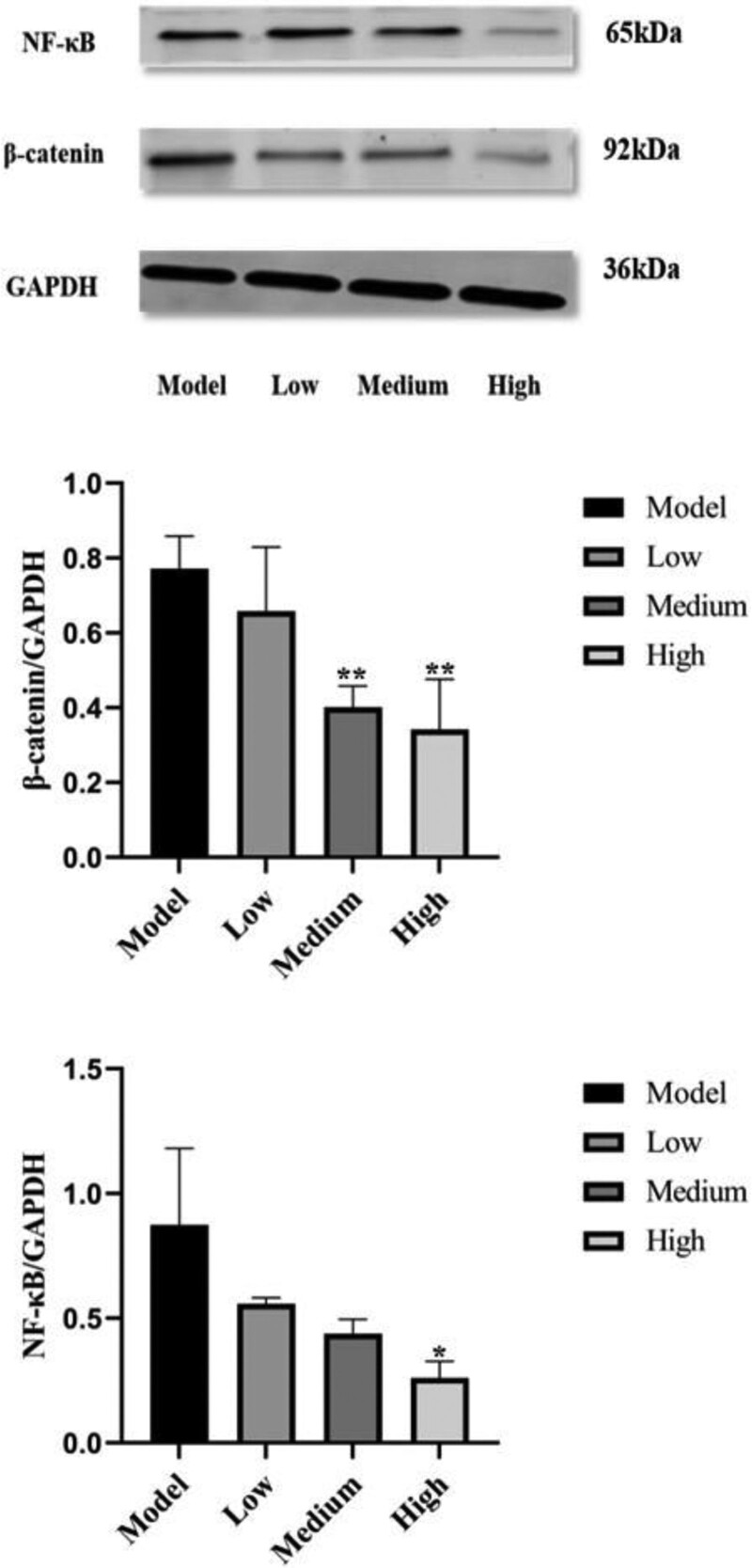
Western blotting was conducted to determine the protein expression of NF-κB p65 and β-catenin.

After the pro-apoptotic effect was confirmed, we went on to explore the molecular mechanism of its action by Western blotting (Fig. 5). The result measured showed that the protein expression of p65 and β-catenin in the XF group (3, 4, 5 mg/mL) was significantly reduced. These results suggested that XF could inhibit the protein expression of factors related to the NF-κB pathway and Wnt/β-catenin pathway.

## 4. Discussion

RA is a chronic and debilitating systemic inflammatory disease. At present, there are more than 21 million patients with rheumatoid arthritis worldwide.^[21]^ The pathological manifestations of RA are complex, affecting the synovium and tissues of joints for a long time, and the course of treatment is long. Over the years, RA has become a serious social and public health problem. FLS could secrete cytokines and proteolytic enzymes, aggravating inflammation and cartilage breakdown.^[22]^ HFLS-RA cells are 1 of the main effector cells during human RA, and their pathological changes play a key role in joint destruction and chronic persistent inflammation.^[23–25]^ HFLS-RA cells have the characteristics of abnormal proliferation and apoptosis in the pathological process of RA,^[26]^ so inhibiting the abnormal proliferation of HFLS-RA cells and inducing apoptosis have become 1 of the main strategies to treat RA.^[27]^ As a common Chinese medicine, XF is often used to treat damp arrest contracture and rubella pruritus. The Chinese Pharmacopoeia (2020 edition) records that XF can dispel wind-cold, relieving stuffy nose and expelling wind and dampness. Modern studies show that XF contains fatty acids, phenolic acids, lignans and other chemical components, and has various pharmacological effects such as anti-inflammatory, analgesic, antibacterial and antiviral.^[28]^ Huang et al found that XF has a strong anti-inflammatory effect.^[29]^

In this study, we observed the influence of XF on the proliferation of HFLS-RA cells in vitro by the MTS method, and found that XF could significantly inhibit the proliferation of HFLS-RA cells in a dose-dependent manner. Hoechst 33,342, a bisbenzimide derivative, can be used for observing the DNA content of living FLS-RA cells. We used Hoechst 33,342 stain and flow cytometry to observe the apoptosis effect of XF, and our results showed that the amount of apoptosis of FLS cells increased after treatment. These findings all suggest that XF has a particular therapeutic effect on RA.

Inflammatory cytokines regulate innate and adaptive immune responses to some extent, and play a key role in the generation of synovitis and the development of RA. In the cytoplasm, the nuclear localization (NLS) region of NF-κB is covered by its inhibitor IκB to prevent nuclear translocation.^[30]^ When stimulated, IκB is phosphorylated and degraded. NF-κB p65 can then move to the nucleus and bind to target genes, which can enhance the expression level of many inflammatory mediators (such as TNF-α, IL-1β, COX2, etc.) expressed in the synovium of rheumatoid arthritis.^[31]^ Furthermore, it has been suggested that NF-κB p65 regulates cell apoptosis and inhibits protein expression, having an antagonistic effect on the apoptosis of FLS-RA cells. Additionally, it was also found that NF-κB p65 promotes the proliferation of synovial cells and inhibits their apoptosis, leading to synovial hypertrophy and proliferation and aggravating the destruction of joint structures.^[32]^ Our study showed that XF significantly reduced NF-κB p65 expression in HFLS-RA cells compared to the model group, indicating that XF could inhibit the inflammatory reaction of RA. The free and stable β-catenin in the cytoplasm can effectively regulate the excessive proliferation and inflammation of fibroblasts by entering the nucleus.^[33]^ The experimental data showed that after XF was given, the content of β-catenin decreased significantly with the increase of dose, suggesting that XF could repress the proliferation of FLS cells. XF has an excellent curative effect on RA.

In summary, XF can inhibit HFLS-RA cells and promote apoptosis in a dose-dependent manner. The results showed that the mechanism by which XF produces this effect is through inhibiting the production of the NF-κB pathway and the Wnt/β-catenin pathway, and the expression levels of NF-κB p65 and β-catenin were significantly decreased. Our results will help clarify the mechanism of XF on RA and provide theoretical support for its clinical application. However, the limitation of this study is that there is no research on experiments in animals in vivo, and the biomarkers of anti-RA need further exploration. In the future, we will continue to study the mechanism and efficacy of XF in treating RA from other aspects, and further study the specific chemical components of XF that are involved in treating RA.

## 5. Conclusion

In conclusion, XF extract significantly inhibited the inflammatory response, prevented proliferation, and promoted apoptosis of HFLS-RA cells. We verified that NF-κB p65 and β-catenin are potential biomarkers related to RA. However, further studies are needed to fully understand the therapeutic mechanism of RA. This study also finds a new effective drug to prevent and treat RA, providing an experimental basis for further development and clinical research of XF.

## Author contributions

**Conceptualization:** Haixue Kuang, Liu Yang.

**Data curation:** Hai Jiang, Huan Yu.

**Funding acquisition:** Hai Jiang, Liu Yang.

**Investigation**: Hai Jiang, Senwang Zheng.

**Methodology**: Hai Jiang.

**Project administration**: Hai Jiang, Xuejiao Wang.

**Resources**: Ajiao Hou.

**Supervision**: Huan Yu.

**Validation**: Hai Jiang.

**Visualization:** Hai Jiang.

**Writing** – **original draft**: Hai Jiang

**Writing** – **review & editing**: Haixue Kuang, Liu Yang.
